# Observed changes in the Earth’s dynamic oblateness from GRACE data and geophysical models

**DOI:** 10.1007/s00190-015-0852-y

**Published:** 2015-09-18

**Authors:** Y. Sun, P. Ditmar, R. Riva

**Affiliations:** Department of Geoscience and Remote Sensing, Delft University of Technology, Delft, The Netherlands

**Keywords:** $J_{2}$, $C_{20}$, Satellite laser ranging, Glacial isostatic adjustment, Temporal gravity field variations, Mass transport

## Abstract

A new methodology is proposed to estimate changes in the Earth’s dynamic oblateness ($\Delta {J_{2}}$ or equivalently, $-\sqrt{5}\Delta {C_{20}}$) on a monthly basis. The algorithm uses monthly Gravity Recovery and Climate Experiment (GRACE) gravity solutions, an ocean bottom pressure model and a glacial isostatic adjustment (GIA) model. The resulting time series agree remarkably well with a solution based on satellite laser ranging (SLR) data. Seasonal variations of the obtained time series show little sensitivity to the choice of GRACE solutions. Reducing signal leakage in coastal areas when dealing with GRACE data and accounting for self-attraction and loading effects when dealing with water redistribution in the ocean is crucial in achieving close agreement with the SLR-based solution in terms of de-trended solutions. The obtained trend estimates, on the other hand, may be less accurate due to their dependence on the GIA models, which still carry large uncertainties.

## Introduction

Monthly Earth gravity field models based on data from the Gravity Recovery and Climate Experiment (GRACE) satellite mission (Tapley et al. 2004), which was launched in 2002, are being released by several data analysis centers (e.g., Center for Space Research (CSR) model RL05 (Bettadpur 2012), Geo Forschungs Zentrum (GFZ) model RL05a (Dahle et al. 2013), Jet propulsion Laboratory model (JPL) RL05 (Watkins and Yuan 2012), Delft Mass Transport model (DMT) (Liu et al. 2010). In spite of continuous improvements in data processing techniques, very low-degree spherical harmonic coefficients still cannot be determined with high accuracy. This is largely due to the mission design (low orbits, limited separation of the satellites, etc.) (Chen et al. 2005). In particular, this concerns variations of the $C_{20}$ coefficient ($\Delta {C_{20}}$, denoted as $C_{20}$ hereafter for simplicity), which describes changes of the Earth’s dynamic oblateness $J_{2}$ ($J_{2} = -\sqrt{5} C_{20}$, where the factor $\sqrt{5}$ implicitly means that the $C_{20}$ is normalised). Estimations of this coefficient are corrupted by 161-day-period ocean tide aliases (Cheng et al. 2013). Therefore, the $C_{20}$ coefficient in GRACE gravity field models is recommended to be replaced with estimates from other techniques such as satellite laser ranging (SLR), which is likely to provide the most accurate $C_{20}$ information so far (Cheng and Tapley 2004).

An alternative source of information about variations of low-degree coefficients is surface mass loading inferred from the GPS-sensed solid Earth deformation, an approach known as the inversion method (Blewitt et al. 2001; Gross et al. 2004; Wu et al. 2012).

Swenson et al. (2008) developed a new method to determine the degree-1 coefficients by combining GRACE information with ocean bottom pressure (OBP) data, so that the usage of GPS data is not needed.

Here, we extend the methodology by Swenson et al. (2008) further to estimate the monthly $C_{20}$ coefficients from other GRACE gravity field model coefficients supported by the $C_{20}$ coefficients from an OBP model and a glacial isostatic adjustment (GIA) model. We validate our solutions against SLR-derived estimates. This study is motivated by the following considerations: (1) dense and evenly distributed measurements are used as the input. (2) The proposed procedure has better prospects regarding an increasing accuracy of future satellite gravity mission and related geophysical models. In addition, one will be able to use the proposed procedure for a mutual validation of the estimates based on GRACE data and on other techniques.

## Methodology

Following equation (11) in Swenson et al. (2008), one can derive a similar equation for the determination of the $C_{20}$ coefficient:

1 $$\begin{aligned} C_{20}^{'} = \frac{4\pi C_{20}^{{\text {ocean}}^{\prime }} - \int \mathrm{d}{\varOmega } \bar{P}_{20}(\cos \theta )\vartheta (\theta ,\phi )\sum \nolimits _{l=1}^{\infty }\sum \nolimits _{m=0}^{l}\bar{P}_{lm}(\cos \theta )\{C_{lm}^{'}\cos m\phi + S_{lm}^{'}\sin m\phi \}}{\int \mathrm{d}{\varOmega } \bar{P}_{20}(\cos \theta )\vartheta (\theta ,\phi )\bar{P}_{20}(\cos \theta )}, \end{aligned}$$

where $C_{20}^\mathrm{ocean'}$ represents the oceanic component of $C_{20}^{'}$. Integrals are defined over the entire globe, $\mathrm{d}{\varOmega }=\sin \theta \mathrm{d}\theta \mathrm{d}\phi$ is an element of solid angle. The summations exclude the estimated term $C_{20}^{'}$. Indices *l* and *m* stand for spherical harmonic degree and order, respectively. $\bar{P}_{lm}$ are normalised associated Legendre functions. $\theta$ is colatitude in spherical coordinates, $\phi$ is longitude, $\vartheta (\theta , \phi )$ denotes the ocean function, which equals 1 over ocean and 0 over land. $C_{20}^{'}$, $C_{lm}^{'}$ and $S_{lm}^{'}$ denote the “mass coefficients” describing the surface mass change and are related to the dimensionless Stokes coefficients $C_{20}$, $C_{lm}$ and $S_{lm}$ by

2 $$\begin{aligned} \left[ \begin{array}{c} C_{lm}^{'} \\ S_{lm}^{'} \end{array}\right] = \frac{a\rho _{\text {earth}}(2l+1)}{3(1+k_{l})} \left[ \begin{array}{c} C_{lm} \\ S_{lm} \end{array} \right] , \end{aligned}$$

in which *a* is the semi-major axis of the reference ellipsoid, $\rho _\mathrm{earth}$ is the Earth’s average density and $k_{l}$ denotes the degree-l load Love number (Wahr et al. 1998).

Following Swenson et al. (2008), one can easily extend equation (1) to the case when four coefficients—$C_{10}^{'}$, $C_{11}^{'}$, $S_{11}^{'}$, and $C_{20}^{'}$—have to be simultaneously estimated, for which purpose a system of linear equations has to be solved:

3 $$\begin{aligned} \left[ \begin{array}{l@{\quad }l@{\quad }l@{\quad }l} I_{10C}^{10C} I_{11C}^{10C} I_{11S}^{10C} I_{20C}^{10C} \\ I_{10C}^{11C} I_{11C}^{11C} I_{11S}^{11C} I_{20C}^{11C} \\ I_{10C}^{11S} I_{11C}^{11S} I_{11S}^{11S} I_{20C}^{11S} \\ I_{10C}^{20C} I_{11C}^{20C} I_{11S}^{20C} I_{20C}^{20C} \end{array}\right] \left[ \begin{array}{c} C_{10}^{'} \\ C_{11}^{'} \\ S_{11}^{'} \\ C_{20}^{'} \end{array}\right]= & {} \left[ \begin{array}{c} C_{10}^\mathrm{ocean'} \\ C_{11}^\mathrm{ocean'} \\ S_{11}^\mathrm{ocean'} \\ C_{20}^\mathrm{ocean'} \end{array}\right] \nonumber \\&- \left[ \begin{array}{c} G_{10C} \\ G_{11C} \\ G_{11S} \\ G_{20C} \end{array}\right] , \end{aligned}$$

where the following notations have been used:

4 $$\begin{aligned} I_{20C}^{11S}=&\frac{1}{4\pi } \int \mathrm{d}{\varOmega } \nonumber \\&\bar{P}_{11}(\cos \theta )\sin (1\times \phi ) \vartheta (\theta ,\phi )\bar{P}_{20}(\cos \theta )\cos ( 0\times \phi )) \nonumber \\&(\hbox {similar for the other elements of matrix { I}}), \end{aligned}$$

and

5 $$\begin{aligned} G_{20C}=&\frac{1}{4\pi } \int \mathrm{d}{\varOmega }\bar{P}_{20}(\cos \theta )\cos (0\times \phi ) \vartheta (\theta ,\phi ) \nonumber \\&\sum \limits _{l=2}^{\infty } \sum \limits _{m=0}^{l}\bar{P}_{lm}(\cos \theta )\{C_{lm}^{'}\cos m\phi +S_{lm}^{'} \sin m\phi \} \nonumber \\&(\hbox {similar for the other elements of vector { G}}), \end{aligned}$$

in which the summations exclude the terms that are estimated.

To solve the system of linear equations and obtain degree-1 and $C_{20}$ dimensionless Stokes coefficients, one needs (1) the oceanic component of degree-1 and $C_{20}$, (2) higher-order Stokes coefficients and (3) GIA model coefficients. The input and output shown in the equation are mass coefficients, but they are directly related to the Stokes coefficients mentioned here through Eq. (2). The Stokes coefficients used in this study come directly from the GRACE level-2 data (also known as GSM), for which the oceanic and atmospheric mass variations are subtracted using the atmosphere and ocean de-aliasing level-1B (AOD1B) products (Flechtner et al. 2014). Monthly averages of the AOD1B product are available in Stokes coefficients stored in GAC and GAD files. GAC includes global oceanic and atmospheric effects, while GAD has the atmospheric contribution over land set to zero. To make the input coefficients compatible, the same oceanic and atmospheric effects need to be removed also from the oceanic coefficients, e.g., $C_{20}^\mathrm{ocean'}$. Since the oceanic coefficients lack the contribution from atmosphere over continents, it is the GAD (rather than GAC) which should be subtracted. With this procedure, the output will also be GSM-like coefficients. If the full $C_{20}$ coefficients are needed, the contribution of GAC can be restored afterwards.

An alternative procedure requires that the AOD1B product is first added back to GSM coefficients and then full degree-1 and $C_{20}$ coefficients are estimated directly. Although the latter procedure is stated to be equivalent to the first one in Swenson et al. (2008), it is not favoured in this study for the reason outlined in Sect. 3.2.

## Input data

### Oceanic $C_{20}^ {'}$

As has been mentioned above, the GAD contribution, denoted as $C_{20}^\mathrm{GAD'}$, needs to be removed from $C_{20}^\mathrm{ocean'}$ coefficients. The GAD coefficients represent the OBP model that describes the pressure on the sea floor from both air and water column above. The water columns are output from the ocean model from circulation and tides (OMCT) (Thomas 2002). This ocean model applies the Boussinesq approximation and thus essentially conserves the ocean volume. A thin uniform layer of water is then added or removed to conserve the total ocean mass. As a result, $C_{20}^\mathrm{GAD'}$ should include the contribution of internal oceanic mass redistribution as well as the atmospheric mass variations over the ocean regions. After removing $C_{20}^\mathrm{GAD'}$, the remaining of $C_{20}^\mathrm{ocean'}$ reflects only the water exchange between ocean and continents ($C_{20}^\mathrm{excange'}$). Therefore, the input $C_{20}^\mathrm{ocean'}$ coefficients are equal to $C_{20}^\mathrm{exchange'}$ in our study in view of the fact that OMCT is exploited as the OBP model.

The aforementioned $C_{20}^\mathrm{exchange'}$ can also be provided by GRACE data (Chambers and Schroeter 2011). In this study, we integrate the GSM coefficients over the continental areas to infer the total water mass variations (which are opposite to the water mass variations in the oceans, assuming mass conservation in the Earth system). Once the monthly water mass variation is known, the value of $C_{20}^\mathrm{exchange'}$ can be obtained by assuming a certain spatial distribution of the exchanged water over the oceans. We implement two different approaches: (1) water redistributes as a uniform layer [eustatic approach, as in Swenson et al. (2008)]; (2) water redistributes accounting for Self-attraction and loading effects (SAL approach). SAL effects [or fingerprints, Mitrovica et al. (2001)] are computed by solving the sea-level equation (Farrell and Clark 1976), including the feedback from Earth rotation (Milne and Mitrovica 1998). It is worth noting that using GRACE to constrain total mass change over the continents requires the availability of a complete GRACE solution, which includes the coefficients being estimated through Eq. (3). Therefore, $C_{20}^\mathrm{exchange'}$ needs to be determined through an iterative approach (starting from a GRACE solution where the four estimated coefficients are null, later updated with preliminary estimates of the same coefficients). Convergence is very quick, with the difference between subsequent solutions being smaller than 0.1 % in 3 or 4 iterations.

The degree-1 coefficients are estimated similarly, simultaneously with $C_{20}$.

### GRACE gravity field models

In this paper, we present results based on CSR RL05, GFZ RL05a and JPL RL05 time series in the period from January 2003 to May 2013, all complete to or truncated at degree 60.

All the GRACE-based monthly gravity fields contain spatially correlated noise that reveals itself in the form of meridionally oriented stripes in the spatial domain. To solve the sea level equation and account for self-attraction and loading effects, we need to know the spatial distribution of the land load as accurately as possible. For this purpose, we use publicly available solutions that have been post-processed by means of the DDK4 filter (Kusche et al. 2009) (http://icgem.gfz-potsdam.de/ICGEM/). The DDK4 filter is a decorrelation filter making use of error covariance matrices, and an a priori signal covariance matrix in the spherical harmonic domain. In this way, the filtering ensures that a higher noise or/and lower signal level means harder damping and vice versa. Ultimately, the effect of this filter is somewhat similar to that of a combination of empirical destriping algorithm (Swenson and Wahr 2006) and Gaussian filter (Wahr et al. 1998).

When using Eq. (3), we need to deal with the limited spatial resolution of the GRACE gravity field models, which causes signals to spread over (or leak into) wider areas. The signal leakage is further increased by applying a filter, such as DDK4. As a result, the available observations cannot distinguish whether mass variations occurring in coastal areas are originating from the land or from the ocean. An attempt to define an ocean function without taking this fact into account may lead to a miscalculation of the total mass exchange between land and oceans as well as of the *G* vector. We correct for signal leakage by introducing a buffer zone around all land areas, similarly to what is done by Swenson et al. (2008) when computing the total ocean mass change. Differently from that study, we also consider the buffer zone to be part of the land areas when we define the ocean function $\vartheta (\theta , \phi )$, which means that we include the buffer zone in the definition of the *G* vector. We will show that such a buffer is crucial to obtain solutions close to SLR estimates. The use of a buffer introduces the risk that mass redistribution due to ocean dynamical processes in coastal areas is erroneously attributed to land processes. However, the problem is largely reduced using GSM coefficients in Eq. (3), under the assumption that the AOD products capture most of the ocean signal.

### GIA models

The method discussed above and Eq. (2) imply that gravity field variations are solely due to a redistribution of mass at the Earth’s surface. Solid Earth contributions such as those of tectonics and GIA should, therefore, be removed. Here, only GIA is accounted for as proposed by Swenson et al. (2008). The removed GIA signal is restored at the final data processing stage. Since GIA is characterised by a linear trend, the choice of a specific GIA model has no impact on seasonal and other short-term signals.

Considered that available GIA models are highly uncertain, we only show the resulting C20 trends for a few GIA realisations, based on different Earth rheologies and on two Antarctic ice histories. A full-scale sensitivity study is beyond the scope of this paper.

Four GIA models have been used in this study. All models are based on the ICE-5G ice history (Peltier 2004). Model-A, -B and -C are based on a simplified version of viscosity model VM2 (Peltier 2004), while Model-D assumes a lower mantle with a higher viscosity ($10^{22}$ Pa s) than VM2 (Mitrovica and Forte 1997). Model-A is taken from A et al. (2012), who computed it for a compressible earth model, while Model-B, -C, and -D are our own realization and make the commonly used assumption of incompressibility within the Solid Earth (Spada et al. 2011). In Model-C, the Antarctic component is computed separately, based on ice history IJ05 (Ivins and James 2005) and on a different viscosity profile than VM2 (consisting of a 60-km-thick elastic lithosphere and of a lower mantle with a viscosity of $10^{22}$ Pa s). This Antarctic setup provides uplift rates very close to independent results based on satellite data (Riva et al. 2009).

## Results

The following factors can affect the estimation of $C_{20}$ coefficients: (1) the choice of the input models (GRACE solutions, OBP and GIA models) and (2) implementation details (buffer zone width, the filter applied to GRACE solutions and whether or not accounting for self-attraction and loading effects). By trying different combinations of data processing parameters, we produced many variants of $C_{20}$ time series. Each of them was compared with the state-of-the-art $C_{20}$ time series based on SLR data from five geodetic satellites (LAGEOS-1 and 2, Starlette, Stella and Ajisai) (Cheng et al. 2013). Since all the results discussed are presented in the form of GSM-like coefficients, the AOD1B product (GAC coefficients) has also been removed from the reference SLR time series. We estimate bias, linear trend, acceleration, as well as annual and semi-annual periodic terms for each time series and make a comparison with corresponding parameters derived from the SLR-based time series.

We first compare de-trended (linear-trend removed) time series both visually and in terms of variance, where the percentage of the SLR variance explained is defined as $R^{2} = 1 - \langle \mathrm{SLR} - \mathrm{MODEL} \rangle / \langle \mathrm{SLR} \rangle$, where MODEL represents our estimation in this study and $\langle \rangle$ denotes the variance operator. We also compare annual amplitudes and phases against those of the SLR solution. Comparison of de-trended time series will lead to results invariant to the GIA model used. Later, we use one selected solution to compare the linear trend estimates resulting from different GIA models.

### Seasonal variations

**Fig. 1:**
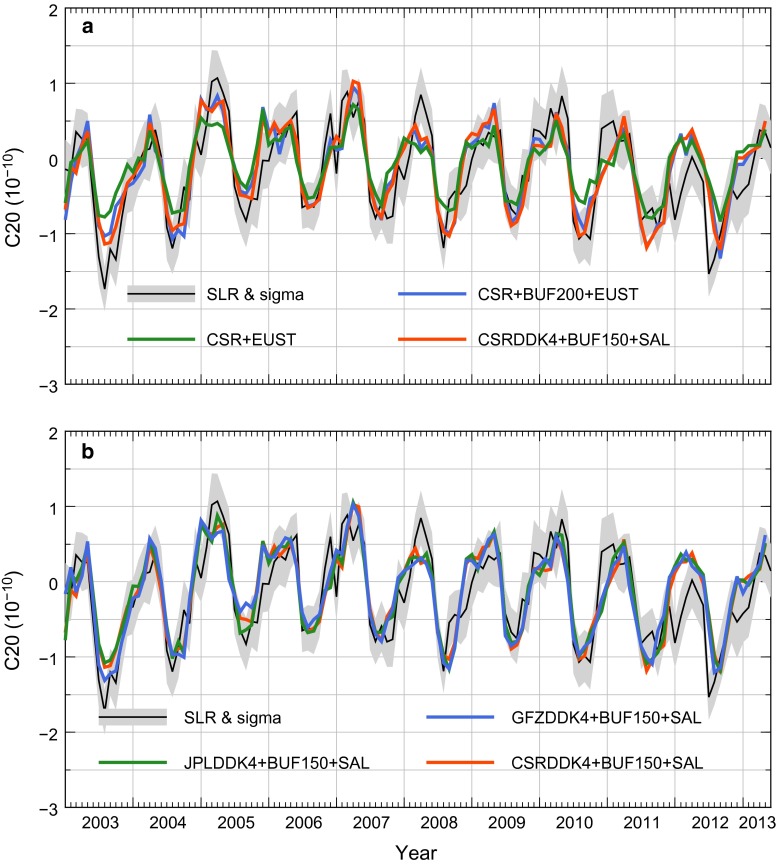
Selected GRACE-based $C_{20}$ solutions obtained using different implementation details and input models, together with an SLR-derived solution and its standard deviation. A linear trend has been removed. **a** The role of implementation details. **b** The effect of using different GRACE solutions after fixing the implementation parameters. The reference SLR solution and its one standard deviation are shown in both panels (*black solid line* and *grey band*). GRACE solution used, buffer zone width (not shown if no buffer zone used) and whether the SAL effects are accounted for are shown in the name of each solution

In Fig. 1, we show a few time series meant to illustrate the sensitivity of our GRACE-based solutions to implementation details and input models. The reference SLR solution is represented by a black solid line and by a grey band, indicating mean value and one standard deviation, respectively. In Table 1, we show statistics for the same models, as well as for a few additional experiments (different buffer widths, use of the DDK4 filter).

In Fig. 1a, we show the role of implementation details, namely of the use of a buffer zone and of the computation of SAL effects, based on GRACE CSR RL05 solutions. Not using any buffer and ignoring SAL effects (green line) largely underestimates the amplitude of the seasonal cycle. Nonetheless, most features of the SLR time series are already recognisable, such as the relative size of maxima and minima, as well as their phase. This solution explains about 59 % of the SLR variance, where the annual cycle is rather close in phase, but clearly smaller in amplitude (65 % of SLR). The addition of a 200 km buffer zone (blue line) largely improves the overall (explained variance) fit as well as the size of the peak amplitudes. The amplitude of the annual signal becomes statistically equivalent (within $2\sigma$) to the SLR solution. However, the improvement on the overall fit is moderate, where the new solution explains about 68 % of the SLR variance. Further increase in the width of the buffer zone to 250 and 300 km will begin to lower the explained variance slightly. When using a 300 km buffer width, the annual amplitude estimated becomes smaller. The analysis of the buffer zone width and more advanced way of handling signal leakage will be discussed in a separate paper.

**Table 1:** Statistics for GSM-like $C_{20}$ time series estimated with different strategies and for different GRACE solutions

	Var. expl. (%)	Trend ($10^{-11}\,\mathrm{year}^{-1}$)	Annual signal	
			Amplitude ($10^{-11}$)	Phase (day)
SLR	100.0	$-1.0 \pm 0.1$	$6.9 \pm 0.4$	$82.0 \pm 3.3$
CSR + EUST	58.6	$+0.1 \pm 0.1$	$4.5 \pm 0.2$	$71.3 \pm 2.8$
CSR + BUF150 + EUST	68.0	$-1.2 \pm 0.1$	$6.1 \pm 0.3$	$76.9 \pm 2.7$
CSR + BUF200 + EUST	68.2	$-1.4 \pm 0.1$	$6.3 \pm 0.3$	$77.5 \pm 2.8$
CSRDDK4 + EUST	57.4	$+0.3 \pm 0.0$	$4.1 \pm 0.2$	$70.1 \pm 2.7$
CSRDDK4 + BUF150 + EUST	68.2	$-0.8 \pm 0.1$	$5.4 \pm 0.2$	$75.5 \pm 2.6$
CSRDDK4 + BUF200 + EUST	69.2	$-1.1 \pm 0.1$	$5.6 \pm 0.3$	$76.3 \pm 2.6$
CSRDDK4 + BUF250 + EUST	68.9	$-1.4 \pm 0.1$	$5.6 \pm 0.3$	$77.4 \pm 2.9$
CSRDDK4 + BUF300 + EUST	67.3	$-1.6 \pm 0.1$	$5.4 \pm 0.3$	$77.2 \pm 3.1$
CSRDDK4 + BUF400 + EUST	61.7	$-1.7 \pm 0.1$	$4.7 \pm 0.3$	$78.3 \pm 4.0$
CSRDDK4 + BUF500 + EUST	59.3	$-1.6 \pm 0.1$	$4.5 \pm 0.3$	$76.6 \pm 4.4$
CSRDDK4 + SAL	61.5	$-0.0 \pm 0.1$	$4.8 \pm 0.2$	$70.5 \pm 2.6$
**CSRDDK4 + BUF150 + SAL**	**70**.**8**	$-\mathbf 1.3 \pm \mathbf 0.1$	$\mathbf 6.8 \pm \mathbf 0.3$	$\mathbf 77.4 \pm \mathbf 2.3$
CSRDDK4 + BUF200 + SAL	70.8	$-1.6 \pm 0.1$	$7.1 \pm 0.3$	$78.3 \pm 2.3$
GFZDDK4 + BUF150 + SAL	71.6	$-1.4 \pm 0.1$	$6.9 \pm 0.3$	$75.2 \pm 2.5$
JPLDDK4 + BUF150 + SAL	70.0	$-1.4 \pm 0.1$	$7.0 \pm 0.3$	$78.8 \pm 2.2$
SLR FULL		$-1.0 \pm 0.2$	$14.2 \pm 0.7$	$52.4 \pm 2.8$

The trend is based on GIA realisation Model-C, where the GIA contribution to the trend has been restored. The solution SLR FULL, where the AOD1B fields have not been removed, is provided as a reference. The highlighted solution (in bold) is recommended and is available online at http://www.citg.tudelft.nl/c20

Finally, accounting for SAL effects (red line) further improves the explained variance and at the same time significantly affects the amplitude of the estimated annual signal. The solution closest to SLR when including SAL effects (the explained variance is 71 %) makes use of a smaller buffer (150 km) than in the eustatic case. Note that the effects of feedback from the Earth rotation are accounted for during the computation of SAL. These effects on the estimated $C_{20}$ coefficients are negligible (not shown).

It is worth mentioning that the elimination of the buffer zone from the ocean functions prevents the accounting for SAL effects in the coastal regions. We have verified, however, that this has a little impact on the solution. We have considered the following two scenarios: (1) solving the sea level equation for the whole ocean; (2) solving the sea level equation for a slightly smaller ocean by reducing the ocean function 150 km along all boundaries while keeping the continental load unchanged (i.e., ignoring the mass variation inside the 150-km-wide buffer zone seen by GRACE). The resulting amplitude of the annual signal in the second scenario increases, compared to the first one, by only about 2 %, which is less than the uncertainty.

In Fig. 1b, we fix the implementation parameters and show the effect of using different GRACE solutions. The GFZ solution provides the best overall fit (71.6 % of the SLR variance explained and same amplitude of the annual signal). Nonetheless, all three time series—GFZ, CSR and JPL—are very close to each other and the amplitude of the annual signal is statistically equivalent (within $1\sigma$).

The phase estimates are not significantly affected by any of the above-mentioned factors. The differences of phase estimates compared to those based on SLR data are all within ten days.

### Trend estimates and GIA

Table 1 also lists linear trend estimates when using GIA model Model-C. Note that those trends are still based on the GSM-like solutions, but we have verified that long-term trends in atmospheric pressure over land and in OBP are negligible. The table shows that both buffer and SAL effects have a large impact on the trend due to the present-day mass transport (PDMT). The estimated trend is zero without buffer and SAL, but eventually becomes 40 % larger than the SLR trend for the model that provides the best fit of the seasonal signal. The largest effect originates from the buffer, but also SAL effects are sizeable (causing a further increase of up to 14 % when the buffer width is 200 km).

In Table 2, we list the effect of using different GIA models for the results based on DDK4-filtered CSR solutions in combination with a 150-km buffer and taking SAL effects into account (i.e., CSRDDK4 + BUF150 + SAL). Similar conclusions hold for other setups. To allow an easier comparison with previous studies, we show the obtained trends in terms of $\dot{J}_2$.

**Table 2:** $J_{2}$ trends estimated using different GIA models (unit $10^{-11}\,\mathrm{year}^{-1}$)

	GIA	PDMT	Total
SLR	$-$	$-$	$2.2 \pm 0.2$
Model-A	$-$3.3	7.4	$4.1 \pm 0.2$
Model-B	$-$3.6	7.1	$3.6 \pm 0.2$
Model-C	$-$3.6	6.6	$3.0 \pm 0.2$
Model-D	$-$5.7	9.1	$3.4 \pm 0.2$

Results are based on solution CSRDDK4 + BUF150 + SAL

**Table 3:** Estimated annual amplitude and phase of global ocean mass variations

Measurement source	Method	Time span	Amplitude (mm)	Phase (day)
CSR + EUST	GRACE	2003–2013	$8.8\pm 0.2$	$285 \pm 2$
CSR + BUF150 + EUST	GRACE	2003–2013	$9.5\pm 0.2$	$280 \pm 1$
CSR + BUF200 + EUST	GRACE	2003–2013	$9.4\pm 0.2$	$279 \pm 1$
CSRDDK4 + EUST	GRACE	2003–2013	$8.5\pm 0.2$	$285 \pm 1$
**CSRDDK4 + BUF150 + EUST**	**GRACE**	**2003**–**2013**	$\mathbf 9.0 \pm \mathbf 0.2$	$\mathbf 279 \pm \mathbf 1$
CSRDDK4 + BUF200 + EUST	GRACE	2003–2013	$9.0\pm 0.2$	$279 \pm 1$
Chambers et al. (2004)	GRACE	2002–2004	$8.4\pm 1.1$	$270 \pm 8$
	Steric-corrected altimetry	2002–2004	$8.5\pm 0.7$	$282 \pm 5$
Wu et al. (2006)	GPS + GRACE + OBP	1993–2004	9.0	238
Rietbroek et al. (2009)	GPS + GRACE + OBP	2003–2007	8.7	247
Wouters et al. (2011)	GRACE	2003–2010	$9.4\pm 0.6$	$280 \pm 6$
Siegismund et al. (2011)	GRACE	2002–2007	8.4	250
	Steric-corrected altimetry	2002–2007	9.7, 9.6, 9.7	229, 232, 223
Hughes et al. (2012)	In-situ OBP measurements	2002–2010	8.5	266
Bergmann-Wolf et al. (2014)	GRACE	2003–2012	$9.8 \pm 0.5$	278

The recommended solution is shown in bold

**Table 4:** Statistics for the three cartesian components of different geocentre motion solutions

	Trend (mm/year)	Annual signal	
		Amplitude (mm)	Phase (day)
X			
SWENSON_TELLUS	$-0.07 \pm 0.01$	$1.26 \pm 0.05$	$97 \pm 3$
SWENSON_SETUP	$-0.08 \pm 0.01$	$1.30 \pm 0.06$	$97 \pm 3$
CSRDDK4 + BUF150 + SAL	$-{0.05} \pm {0.02}$	${1.50} \pm {0.06}$	${96} \pm {3}$
Y			
SWENSON_TELLUS	$-0.02 \pm 0.02$	$1.50 \pm 0.07$	$-76 \pm 3$
SWENSON_SETUP	$-0.03 \pm 0.02$	$1.42 \pm 0.07$	$-79 \pm 3$
CSRDDK4 + BUF150 + SAL	$+{0.02} \pm {0.02}$	${1.67} \pm {0.07}$	$-{72} \pm {2}$
Z			
SWENSON_TELLUS	$-0.19 \pm 0.02$	$1.77 \pm 0.07$	$92 \pm 2$
SWENSON_SETUP	$-0.20 \pm 0.01$	$1.73 \pm 0.06$	$92 \pm 2$
CSRDDK4 + BUF150 + SAL	$-{0.35} \pm {0.02}$	${2.48} \pm {0.08}$	${88} \pm {2}$

SWENSON_TELLUS has been downloaded from the Tellus website; SWENSON_SETUP uses the same setup as SWENSON_TELLUS, but it results from the simultaneous estimation of $C_{20}$; CSRDDK4 + BUF150 + SAL is the setup that provides the best agreement to SLR-derived $C_{20}$, for the same GRACE solutions and GIA model (Model-A). The GIA contribution to the trend is not restored, as in the SWENSON_TELLUS case

The use of GIA models allows us to separate the contribution of GIA from that of PDMT. The GIA contribution is uniquely defined for each model, while the PDMT value depends on the full GIA spectrum and is, therefore, affected by implementation details.

The smallest (in absolute value) $\dot{J}_2$ of GIA comes from the model by A et al. (2012) (Model-A) which at the same time produces a relatively large estimate for the contribution of PDMT, leading to a larger $\dot{J}_2$ value than the model Model-B based on an incompressible earth. Substituting the Antarctic contribution of ICE-5G with results based on IJ05 (Model-C) has no impact on $\dot{J}_2$ of GIA alone, likely due trade offs between the different ice history and the different viscosity structure used for the Antarctic model. However, the use of IJ05 does reduce the mass loss estimate from Antarctica, leading to a smaller PDMT contribution and to the smallest total $\dot{J}_2$. A higher viscosity in the lower mantle (Model-D) leads to larger contributions from both GIA and PDMT, which compensate each other and result in the second smallest total $\dot{J}_2$.

None of the GIA models tested here provides a very good fit to the $\dot{J}_2$ value determined from SLR. However, our results show positive sign of $\dot{J}_2$, confirming the findings from earlier studies on the inversion of $\dot{J}_2$ observed since 1998 (Cox and Chao 2002), which has been attributed to an increased contribution from PDMT (Dickey et al. 2002; Cheng and Tapley 2004; Nerem and Wahr 2011; Cheng et al. 2013).

### Eustatic sea-level variability and geocentre motion

Finally, it is worth having a brief look at two byproducts of our study: the solutions for eustatic sea-level variability (see Table 3) and for geocentre motion simultaneously obtained with $C_{20}$ (see Table 4).

The eustatic sea-level variability estimated using the approach described in Sect. 3.1 has been compared with recent results based on alternative methods and technologies (Chambers et al. 2004; Rietbroek et al. 2009; Wouters et al. 2011; Siegismund et al. 2011; Hughes et al. 2012; Bergmann-Wolf et al. 2014). Our results are in line with those estimates in terms of annual amplitude and phase.

The co-estimated geocentre motion is significantly different from the one derived from the degree-1 coefficients published on the Tellus website (ftp://podaac.jpl.nasa.gov/allData/tellus/L2/degree_1), both in terms of a trend and annual amplitudes, especially for the *Z*-component. However, the obtained results are statistically equivalent to those published in the Tellus website when we use the same setup as Swenson et al. (2008), where a 300 km buffer zone is used to reduce the signal leakage when estimating the total ocean mass variation, but no buffer zone is considered when define the ocean function. This leaves the question of the optimal estimation of geocenter motion somewhat open. A more thorough analysis of this issue will be the subject of a separate study.

## Discussion and conclusions

Our results (available at http://www.citg.tudelft.nl/c20) show that GRACE data at higher spherical harmonic degrees are capable of estimating seasonal changes in $C_{20}$ to a level comparable with SLR solutions. In fact, the uncertainty (computed as formal error from an analysis of time series) in the amplitude of the annual cycle is smaller for the GRACE-based solutions. This is an indication that our solutions may be less noisy than the SLR one, though it may also imply an underestimation of the signal not described by the fitted curve.

The main factor controlling the amplitude of the seasonal signal is the way how the problem of signal leakage in coastal areas is dealt with. Our simple approach of extending the land mask to include the first few hundreds of kilometres of coastal waters is already capable of producing a solution in close agreement with SLR, though more advanced techniques (e.g., based on mascons) could provide a better way to improve the spatial resolution of GRACE monthly fields and avoid the use of a buffer zone.

Accounting for self-attraction and loading effects driven by the redistribution of continental water masses has the effect of significantly increasing the amplitude of both annual signal and trend.

So far, we have discussed only estimates without the contribution of atmospheric and oceanic processes, assuming that the AOD1B products are correct. In the bottom line of Table 1, we list the full values determined from the SLR time series prior to the subtraction of the AOD1B signal. Compared to the GSM-like solution in the top line, the amplitude of the annual signal is twice as large and its phase is shifted by a month. This suggests that only about half of the seasonal total $C_{20}$ signal is determined by land hydrological processes, including the cryosphere. Therefore, if the proposed methodology is used in estimating the total $C_{20}$ signal, the accuracy of the obtained estimates will be dependent on the accuracy of the atmosphere–ocean model.

The determination of a long-term trend requires the use of a model of GIA, which still carries large uncertainties of an unknown magnitude. Further investigations are warranted in the future to mitigate the uncertainties introduced by a GIA model.

One need to bear in mind that the SLR solution is not free of systematic errors and noise. The processing parameters tuned to achieve a time series that best fit the SLR solution may, therefore, be biased. Further study for validation using accurate geophysical models may enable us to claim an even better solution than that from SLR.
